# Improved adipose tissue function with initiation of protease inhibitor-only ART

**DOI:** 10.1093/jac/dkw301

**Published:** 2016-08-11

**Authors:** Robert T. Maughan, Eoin R. Feeney, Emilie Capel, Jacqueline Capeau, Pere Domingo, Marta Giralt, Joep M. A. Lange, Praphan Phanuphak, David A. Cooper, Peter Reiss, Patrick W. G. Mallon

**Affiliations:** 1 HIV Molecular Research Group, School of Medicine, University College Dublin, Dublin, Ireland; 2 Sorbonne Universities, UPMC Univ Paris 06, INSERM UMRS 938, Paris, France; 3 Infectious Diseases Unit, Hospital de la Santa Creu/Sant Pau, Barcelona, Spain; 4 Department of Biochemistry and Molecular Biology, University of Barcelona, Barcelona, Spain; 5 CIBER Fisiopatologia de la Obesidad y Nutrición, Barcelona, Spain; 6 Academic Medical Center, Department of Global Health and Division of Infectious Diseases, and Amsterdam Institute for Global Health and Development, Amsterdam, The Netherlands; 7 The HIV-Netherlands Australia Thailand Research Collaboration (HIV-NAT) - Thai Red Cross Aids Research Center (TRCARC), Bangkok, Thailand; 8 Department of Medicine, Chulalongkorn University, Bangkok, Thailand; 9 Kirby Institute, University of New South Wales, Sydney, Australia

## Abstract

**Objectives:**

Use of ART containing HIV PIs has previously been associated with toxicity in subcutaneous adipose tissue (SAT), potentially contributing to the development of lipodystrophy and insulin resistance. However, the effect of PIs on SAT function in ART-naive patients independent of other ART classes is unknown. This study aimed to elucidate the effect of initiating PI-only ART on SAT function in ART-naive subjects.

**Methods:**

In the HIVNAT-019 study, 48 HIV-infected, ART-naive Thai adults commencing PI-only ART comprising lopinavir/ritonavir/saquinavir for 24 weeks underwent assessments of fasting metabolic parameters and body composition. In a molecular substudy, 20 subjects underwent SAT biopsies at weeks 0, 2 and 24 for transcriptional, protein, mitochondrial DNA (mtDNA) and histological analyses. ClinicalTrials.gov registration number: NCT00400738.

**Results:**

Over 24 weeks, limb fat increased (+416.4 g, *P *= 0.023), coinciding with larger adipocytes as indicated by decreased adipocyte density in biopsies (−32.3 cells/mm^2^, *P *= 0.047) and increased mRNA expression of adipogenesis regulator *PPARG* at week 2 (+58.1%, *P *= 0.003). Increases in mtDNA over 24 weeks (+600 copies/cell, *P *= 0.041), decreased *NRF1* mRNA expression at week 2 (−33.7%, *P *< 0.001) and increased COX2/COX4 protein ratio at week 24 (+288%, *P *= 0.038) indicated improved mitochondrial function. Despite decreased *AKT2* mRNA at week 2 (−28.6%, *P *= 0.002) and increased *PTPN1* mRNA at week 24 (+50.3%, *P *= 0.016) suggesting insulin resistance, clinical insulin sensitivity [by homeostasis model assessment (HOMA-IR)] was unchanged.

**Conclusions:**

Initiation of PI-only ART showed little evidence of SAT toxicity, the changes observed being consistent with a return to health rather than contributing to lipodystrophy.

## Introduction

Metabolic comorbidities including lipodystrophy, insulin resistance and dyslipidaemia are prevalent amongst people living with HIV (PLWH),^1^ and not only affect adherence to therapy but also contribute to cardiovascular disease risk, a leading cause of death in treated PLWH.^2^ These conditions are in part caused by subcutaneous adipose tissue (SAT) toxicity due to HIV infection and exposure to certain classes of ART.

Although toxicity in SAT has been demonstrated in untreated HIV infection,^3,4^ the development of clinical lipodystrophy (peripheral lipoatrophy, central lipohypertrophy, dyslipidaemia and insulin resistance) is associated with ART exposure,^5^ and once established is not fully reversible.^6^ Lipodystrophy was first attributed to HIV PIs;^7^ however, subsequent studies demonstrated that treatment with thymidine analogue NRTIs (tNRTIs) is the main factor,^8^ primarily via initiation of SAT mitochondrial toxicity.^9^ Due to the routine use of PIs in combination with NRTIs, their relative contribution to ART-mediated SAT toxicity remains unclear.

*In vitro*, PIs inhibit adipogenesis,^10,11^ alter lipid metabolism,^12^ impair glucose uptake insulin signalling,^13,14^ induce mitochondrial dysfunction^11^ and alter secretion of adipocyte-derived hormones and inflammatory cytokines.^12,15^ In clinical studies, PLWH receiving PI-containing ART had reduced expression of key genes required for the regulation of lipid metabolism, insulin sensitivity and adipogenesis in SAT.^16–18^ However, the concurrent use of tNRTIs, as well as the established lipodystrophy, in many of the subjects makes it difficult to determine the specific effects of PIs in these studies.

As no study to date has explored the effects of PI therapy in the absence of other ART classes on SAT function, we aimed to elucidate the effect of initiating PI-only ART through a comprehensive prospective analysis measuring clinical metabolic parameters in parallel with histological and molecular assessments of adipogenic, lipid metabolism, insulin signalling and mitochondrial function pathways in the SAT of ART-naive subjects.

## Methods

### Ethics

The study was approved by the Ethics Committee of the Faculty of Medicine, Chulalongkorn Hospital (approval number 235/2004), written informed consent was obtained for all patients prior to their inclusion and all study procedures were in accordance with standard ethical guidelines.^19^ ClinicalTrials.gov registration number: NCT00400738.

### Study design

A molecular substudy was performed within the HIVNAT-019 trial, an open-label, randomized trial examining the virological efficacy and pharmacodynamics of four different dosing schedules of PI-only ART containing lopinavir boosted with ritonavir co-administered with saquinavir in ART-naive, HIV-1-infected subjects over 24 weeks. Dosing schedules comprised 400/100 mg of lopinavir/ritonavir with 1000 mg of saquinavir twice daily; 400/100 mg of lopinavir/ritonavir with 400 mg of saquinavir twice daily; 266/66 mg of lopinavir/ritonavir with 1000 mg of saquinavir twice daily; and 266/66 mg of lopinavir/ritonavir with 400 mg of saquinavir twice daily. Eligible adult (>18 years), HIV-1 antibody-positive and ART-naive subjects were recruited from the Thai Red Cross Society's Anonymous Clinic and the HIV Outpatient Immune Clinic of King Chulalongkorn Memorial Hospital in Bangkok, Thailand. Relevant inclusion/exclusion criteria and main clinical outcomes were described previously.^20^

### Body composition and fasting blood parameters

Limb and trunk fat were quantified by DXA (Discovery W, Hologic) at weeks 0, 12 and 24. Abdominal subcutaneous and visceral adipose tissue areas at the fourth lumbar vertebra along with SAT at the right mid-thigh were quantified using single-slice CT (Aquilion ONE, Toshiba), described previously.^21^

Fasting bloods (overnight) were drawn for total, LDL and HDL cholesterol, triglycerides, insulin and glucose at weeks 0, 12 and 24. Insulin resistance was estimated using the homeostasis model assessment (HOMA-IR).^22^

### SAT biopsies

Molecular substudy subjects underwent biopsies of abdominal flank SAT as described previously at weeks 0, 2 and 24.^23^ Biopsied tissue was immediately aliquoted and snap frozen in liquid nitrogen for DNA, RNA and protein extraction, and a tissue sample was formalin fixed for histology.

### DNA and RNA extraction from adipose tissue

DNA was extracted from SAT using the QIAamp DNA Mini Kit (Qiagen) and RNA was extracted from homogenized SAT using TRIreagent (Ambion) according to the manufacturer's instructions. The resulting RNA was treated with RQ1 DNase (Promega) and purified using the RNeasy Mini Kit (Qiagen).

### Gene expression

Expression of 55 chosen mRNA targets was determined using a quantitative PCR (qPCR) array (RealTime Ready, Roche), with array details outlined in Table S1 and Table S2 (available as Supplementary data at *JAC* Online). In brief, cDNA libraries were prepared using the Transcriptor First Strand cDNA Synthesis Kit (Roche) and sample quality was verified by qPCR measurement of reference gene actin β (*ACTB*). cDNA libraries underwent a linear 12-cycle pre-amplification using the RealTime Ready cDNA Pre-Amp System (Roche) and then assayed in duplicate with the appropriate controls on the LightCycler 480 (Roche). Gene expression was normalized to the average of reference genes *ACTB*, ribosomal protein L13a (*RPL13A*) and TATA box binding protein (*TBP*).

### Mitochondrial DNA (mtDNA) content

Adipose tissue mtDNA copy number per cell was quantified as described previously.^24^ In brief, DNA samples were quantified against a standard curve of known copy number by qPCR (LightCycler 480, Roche) with primers targeting mitochondrially encoded cytochrome *b* (*MT-CYB*; region 1) and mitochondrially encoded cytochrome *c* oxidase I (*MT-CO1*; region 2). mtDNA levels were compared with nuclear genome DNA copy number with primers targeting peroxisome proliferator-activated receptor γ (*PPARG*) and mtDNA copies/cell was calculated as copy number of mtDNA/(copy number of nuclear DNA/2).

### Protein expression

SAT samples were homogenized in extraction buffer [10 mM HEPES, pH 7.5/5 mM EDTA/5 mM dithiothreitol/5 mM MgCl_2_/PI (Complete Mini, Roche)]. Protein was analysed by immunoblot as previously described,^25^ using antibodies against cytochrome *c* oxidase subunit 2 and 4 (COX2 and COX4) (A-6404 and A-21347, Invitrogen); PPARG and sterol regulatory element binding transcription factor 1 (SREBP1) (Sc-1984X and Sc-367X, respectively, Santa Cruz Biotechnology); and β2-microglobulin (B2M) (P0163, Dako Cytomation). Chemiluminescence was developed using horseradish peroxidase-conjugated secondary antibodies (170-6510, Bio-Rad and 711-135-152, Jackson Immunoresearch) and Immobilon ECL Plus kit (Millipore). ODs were quantified using the Multigauge 3.0 software suite (Fujifilm) and normalized to total protein content.

### Adipose tissue adipocyte density

Paraffin-embedded SAT was cut into 3 μm sections and stained with haematoxylin phloxine saffron by standard protocols.^16^ The number of adipocytes per field (×10 magnification) was quantified using Mercator software (Explora Nova) with adipocyte density expressed as the number of adipocytes/mm^2^.

### Statistical analysis

Subjects with more than one biopsy available were included in molecular analyses. Changes in gene and protein expression were compared using Wilcoxon signed rank tests. Parameters expected to have continuous longitudinal change (mtDNA, adipocyte density and clinical parameters) were analysed using longitudinal marginal models with appropriate covariance structures selected using Akaike's information criterion (AIC). Data are reported as mean (SEM) for marginal model analyses or as median (IQR) otherwise. *P* values <0.05 were considered significant. Gene expression analyses were adjusted for multiple comparisons using the Benjamini–Hochberg procedure.^26^ As the HIVNAT-019 molecular substudy was exploratory, there were no data to guide sample size. However, previous studies demonstrate significant longitudinal reductions in mtDNA and changes in SAT mRNA levels with ART exposure in sample sizes of 20.^23,24^ Statistical analyses were performed using SAS version 9.3 (SAS Institute Inc.) and SPSS statistics V20 (IBM).

## Results

Between October 2004 and March 2006, 20 of 48 subjects randomized to the main study participated in the molecular substudy. Baseline characteristics of substudy subjects were broadly comparable to those of the main study (Table 1), with both genders represented and average baseline CD4+ counts suggesting advanced immunosuppression (14 of 20 subjects in the molecular substudy had a CD4+ T cell count <200 cells/mm^3^).

**Table 1.** Baseline characteristics of study participants

	HIVNAT study	Molecular substudy
*N*	48	20
Male, *n* (%)	20 (42)	11 (55)
Asian, *n* (%)	48 (100)	20 (100)
Age (years), median (IQR)	36 (31.7, 43.3)	39 (30.7, 43.2)
BMI (kg/m^2^), median (IQR)	21.5 (19.4, 24.3)	21.8 (19.5, 23.2)
CD4+ T cell count (cells/mm^3^), median (IQR)	113.5 (67.8, 193.5)	119 (77.5, 217.3)
HIV RNA (log_10_ copies/mL), median (IQR)	4.9 (4.6, 5.1)	4.8 (4.6, 5)

HIV RNA, HIV viral load.

Of the substudy subjects, one was lost to follow-up (week 1), two withdrew for personal reasons (weeks 13 and 21) and one required ART intensification with the addition of NRTI. In the main study, one hepatitis B virus-co-infected subject stopped following a hepatic grade 4 transaminase elevation (week 5) and one required intensification with the addition of NRTI (week 6).

### Changes in clinical metabolic parameters

Over 24 weeks, no changes were observed in BMI or trunk fat by DXA, but limb fat increased significantly [+416.4 (176.1) g, *P *= 0.023], as is expected with ART initiation (Table 2).^5,27^ However, the trunk:limb fat ratio did not change, suggesting a generalized gain in adiposity with ART initiation. Consistent with this, both abdominal subcutaneous and visceral adipose tissue area by CT also increased [+9.5 (4.5) cm^2^, *P *= 0.039 and +4.3 (2.2) cm^2^, *P *= 0.058, respectively], with no significant change in their ratio (Table 2). CT evaluation of thigh SAT revealed a non-significant increase.

**Table 2.** Changes in metabolic parameters over 24 weeks

	Week 0	Week 24	*P* value
Body composition	*n *= 42	*n *= 34	
BMI (kg/m^2^)	21.48 (0.49)	22.1 (0.64)	0.607
total limb fat (kg)	6.9 (0.52)	7.17 (0.57)	0.023
trunk fat (kg)	5.62 (0.42)	6 (0.52)	0.271
trunk:limb fat ratio	0.835 (0.031)	0.835 (0.039)	0.969
abdominal SAT (cm^2^)	128.51 (11.91)	133.83 (12.54)	0.039
abdominal VAT (cm^2^)	39.31 (6.03)	44.66 (8.73)	0.058
abdominal VAT:SAT ratio	0.313 (0.038)	0.329 (0.053)	0.297
thigh SAT (cm^2^)	60.98 (5.89)	66.06 (5.91)	0.102
Fasting parameters	*n *= 48	*n *= 45	
total cholesterol (mmol/L)	4.44 (0.16)	5.52 (0.23)	<0.001
LDL cholesterol (mmol/L)	2.41 (0.13)	2.91 (0.18)	<0.001
HDL cholesterol (mmol/L)	1.29 (0.06)	1.51 (0.06)	<0.001
total cholesterol:HDL cholesterol ratio	3.64 (0.15)	3.86 (0.18)	0.08
triglycerides (mmol/L)	1.3 (0.11)	2.29 (0.26)	0.001
glucose (mmol/L)	4.46 (0.07)	4.73 (0.25)	0.398
insulin (pmol/L)	53.25 (4.96)	49.74 (4.84)	0.399
HOMA-IR	1.57 (0.17)	1.66 (0.28)	0.619

VAT, visceral adipose tissue.

Data are presented as mean (SEM).

*P* values correspond to the output of the marginal model analysis.

Fasting total, LDL and HDL cholesterol and triglycerides all increased, with no significant change in the total cholesterol:HDL cholesterol ratio. No changes were observed in fasting glucose, insulin or HOMA-IR (Table 2).

### Gene expression analysis

The 55 genes examined reflected the following functions: (i) lipid metabolism and adipogenesis; (ii) insulin signalling; (iii) markers of inflammation and cell stress; (iv) adipocyte-secreted hormones; and (v) mitochondrial function. The full list of genes and their corresponding change in expression at weeks 2 and 24 are summarized in Table S1 and Table S3, respectively. Overall, biopsies from 15 subjects contributed to gene expression analyses, with 13 and 12 paired samples for week 2 and week 24 analyses, respectively; 1 sample in the week 24 analysis did not pass initial quality checks.

At week 2, expression of 13 genes changed significantly, 8 of which remained significant after correction for multiple comparisons. Of these 13 genes, 6 were related to lipid metabolism and adipogenesis, 3 to insulin signalling, 2 to mitochondrial function and 2 to cell stress markers. At week 24, only 3 target genes had significantly altered expression, none of which remained significant after correction. Two of these were related to insulin signalling and one to mitochondrial function. The smaller number of differentially expressed genes at week 24 was due to a higher level of variance in expression levels at this timepoint.

### Adipogenesis and lipid metabolism genes

**Figure 1.**
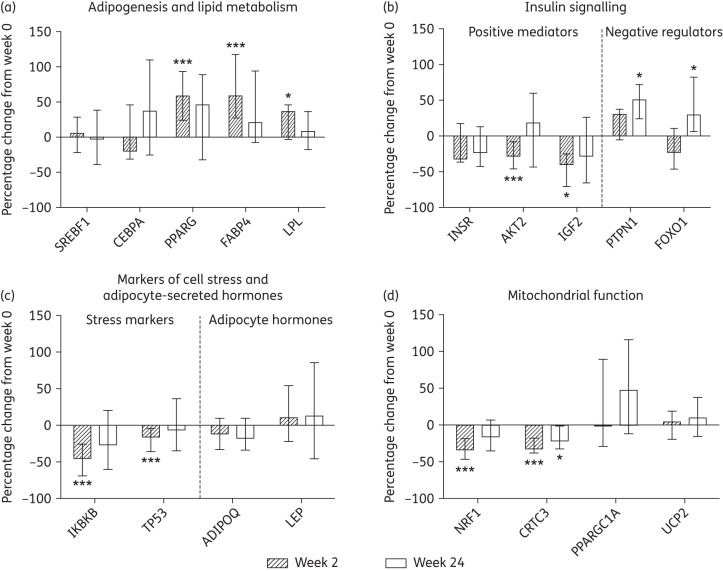
Gene expression changes in the adipose tissue of subjects initiating HIV PI-only ART. Changes in the expression of genes related to (a) adipogenesis and lipid metabolism, (b) insulin signalling, (c) markers of stress and adipocyte-secreted hormones and (d) mitochondrial function were measured by qPCR array. Target gene expression was normalized to the average crossing point of the reference genes: actin β (*ACTB*), ribosomal protein L13a (*RPL13A*) and TATA box binding protein (*TBP*). The bars represent the median (IQR) percentage change from week 0 to week 2 and to week 24; *n* = 13 at week 2 and *n* = 12 at week 24. Statistically significant differences from baseline are labelled *P ≤ 0.05 and ***P ≤ 0.005 (Wilcoxon signed rank test).

The initiation of PI-only ART resulted in significant increases in expression of *PPARG*, the master transcriptional regulator of adipogenesis, at week 2 [+58.1% (+23.5, +93.4), *P *= 0.003, Figure 1a], with a persistent but non-significant increase at week 24 [+45.4% (−32.2, +88.6), *P *= 0.077, Figure 1a]. This was accompanied by increases in week 2 expression (but not week 24) of the downstream PPARG transcriptional targets involved in lipid metabolism: fatty acid binding protein 4 (*FABP4*) and lipoprotein lipase (*LPL*) [+58.8% (+27.3, +117.3), *P *< 0.001 and +36.3% (−2.9, +45.8), *P *= 0.047, respectively, Figure 1a]. Increases in *PPARG* expression and activity indicate that initiation of PI-only ART facilitated increases in SAT lipid metabolism and adipogenesis from diminished baseline levels in ART-naive subjects despite previous associations of PIs with impaired adipogenesis and lipid metabolism.^11,12^ In addition, expression of CCAAT/enhancer binding protein α (*CEBPA*) and sterol regulatory element binding transcription factor 1 (*SREBF1*), major transcriptional regulators of adipogenesis implicated in ART-mediated adipose tissue toxicity,^11,16^ did not change (Figure 1a).

The following were also detected at week 2: (i) decreases in hormone-sensitive lipase (*LIPE*) expression, a key enzyme involved in the triglyceride hydrolysis in adipocytes; (ii) decreases in nuclear receptor coactivator 1 (*NCOA1*), a transcriptional coactivator implicated in adipose tissue energy balance;^28^ and (iii) increases in α-2-glycoprotein 1, zinc-binding (*AZGP1*), a PPARG transcriptional target^29^ (Table 3).

**Table 3.** Overview of differentially expressed genes

Symbol	Gene name	Percentage change	*P* val.	Adj. *P*
Week 0 versus week 2				
*PPARG*	peroxisome proliferator-activated receptor γ	+58.1 (+23.5, +93.4)	0.003	0.026
*FABP4*	fatty acid binding protein 4, adipocyte	+58.8 (+27.3, +117.3)	<0.001	0.009
*LPL*	lipoprotein lipase	+36.3 (−2.9, +45.8)	0.048	0.194
*LIPE*	lipase, hormone-sensitive	−41 (−71.5, −18.1)	0.005	0.031
*AZGP1*	α-2-glycoprotein 1, zinc-binding	+56.3 (+29.6, +107.2)	0.017	0.084
*NCOA1*	nuclear receptor coactivator 1	−14.3 (−33.3, −7.2)	0.012	0.066
*AKT2*	V-akt murine thymoma viral oncogene homologue 2	−28.6 (−46.8, −8.5)	0.002	0.026
*IGF2*	insulin-like growth factor 2 (somatomedin A)	−39.2 (−66.9, −25.7)	0.011	0.063
*RHOQ*	ras homologue family member Q TC10	+42 (+27.2, +74.2)	0.022	0.097
*IKBKB*	inhibitor of κ light polypeptide gene enhancer in B-cells, kinase β	−45.5 (−68.9, −25.8)	<0.001	0.005
*TP53*	P53 tumour suppressor	−15.9 (−36, −4.1)	0.003	0.026
*NRF1*	nuclear respiratory factor 1	−33.7 (−46.9, −18.4)	<0.001	0.005
*CRTC3*	CREB regulated transcription coactivator 3	−32.3 (−38.1, −17.8)	<0.001	0.009
Week 0 versus week 24				
*PTPN1*	protein tyrosine phosphatase, non-receptor type 1	+55.6 (+24, +71.9)	0.024	0.509
*FOXO1*	forkhead box O1	−22 (−32.4, −1.8)	0.027	0.509
*CRTC3*	CREB regulated transcription coactivator 3	+29.1 (+5.7, +82.2)	0.028	0.509

*P* val., *P* values corresponding to comparisons made using Wilcoxon signed rank tests; Adj. *P*, adjusted *P* values corrected for multiple comparisons using the Benjamini–Hochberg procedure.

Data are presented as median (IQR).

Genes are ordered in functional categories corresponding to when they appear in text.

### Insulin signalling genes

*In vivo*, short-term exposure to certain PIs can reduce insulin sensitivity.^30^ Supporting this, changes in gene expression suggested an inhibition of insulin signalling in SAT with PI-only ART initiation. Expression levels of V-akt murine thymoma viral oncogene homologue 2 (*AKT2*), a key transducer of insulin signalling, and insulin-like growth factor 2 (*IGF2*), which activates insulin signalling via the insulin or IGF receptors, were both significantly reduced at week 2 [−28.6% (−46.8, −8.5), *P *= 0.002 and −39.2% (−66.9, −25.7), *P *= 0.011, respectively, Figure 1b], while expression of the insulin receptor (*INSR*) decreased non-significantly [−33% (−36.9, +16.7), *P *= 0.094, Figure 1b]. In addition, expression of two negative regulators of insulin signalling increased: protein tyrosine phosphatase, non-receptor type 1 (*PTPN1*) at both week 2 and 24 [+30.5% (+6.5%, +43.5%), *P *= 0.052 and +55.6% (+22.6, +72), *P *= 0.024, respectively, Figure 1b] as well as forkhead box O1 (*FOXO1*) at week 24 [+29.1% (+5.7, +82.2), *P *= 0.028, Figure 1b].

In contrast, expression of ras homologue family member Q (*RHOQ*) involved in insulin-activated glucose uptake increased significantly at week 2 [+42% (+27.2, +74.2), *P *= 0.022, Table 3].

### Genes encoding markers of cell stress and adipocyte-secreted hormones

Elevated levels of cell stress and inflammation markers along with decreases in adipocyte-secreted hormones are features of ART-mediated SAT toxicity.^16–18^ However, in this study gene expression changes indicated a reduction in cellular stress inflammation with initiation of PI-only ART. Expression levels of tumour protein 53 (*TP53*), a major stress-responsive activator of apoptosis, and IκB kinase β (*IKBKB*), a key activator of inflammatory signalling, were reduced at week 2 [−15.9% (−36, −4.1), *P *= 0.003 and −45.5% (−68.9, −25.8), *P *< 0.001, respectively, Figure 1c]. The expression of other inflammation genes, including *TNF* and *IL6*, did not change significantly, and no changes were detected in the five target genes encoding adipocyte-secreted hormones, including adiponectin (*ADIPOQ*) and leptin (*LEP*) (Figure 1c and Table S3).

### Mitochondrial function genes and mtDNA

Both HIV infection itself and exposure to ART (principally tNRTIs) are associated with altered expression of mitochondrial genes^4,18^ and reduced mtDNA content in SAT.^3,24^ In contrast, the initiation of PI-only ART led to gene expression changes suggesting improvements in mitochondrial function; nuclear respiratory factor 1 (*NRF1*) and CREB regulated transcription coactivator 3 (*CRTC3*), both involved in mitochondrial response to cellular and oxidative stress, were significantly reduced at week 2 [−33.7% (−46.9, −18.4), *P *< 0.001 and −32.3% (−38.1, −17.8), *P *< 0.001, respectively, Figure 1d] and persisted to week 24, significantly so for *CRTC3* [−22% (−32.4, −1.8), *P *= 0.027, Figure 1d]. No accompanying changes were observed in the expression of the key regulator of mitochondrial biogenesis PPARG coactivator 1α (*PPARGC1A*) or in the mitochondrially associated uncoupling protein 2 (*UCP2*) (Figure 1d), genes dysregulated in tNRTI-mediated SAT toxicity.^31,32^

**Figure 2.**
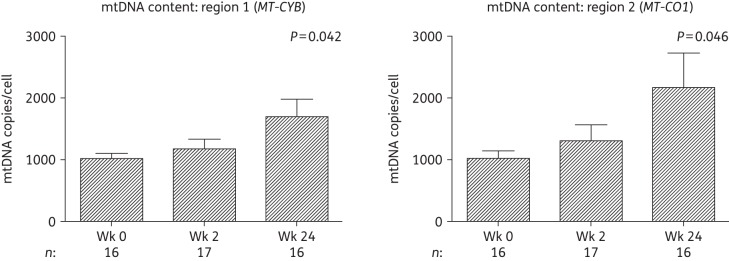
Changes in adipose tissue mtDNA content over 24 weeks in subjects initiating HIV PI-only ART. mtDNA was measured at two regions of the mitochondrial genome by qPCR: mitochondrially encoded cytochrome *b* (*MT-CYB*; region 1) and mitochondrially encoded cytochrome *c* oxidase I (*MT-CO1*; region 2). mtDNA levels were normalized to nuclear genome DNA copy number (primers targeting peroxisome proliferator-activated receptor γ), with mtDNA copies/cell calculated as copy number of mtDNA/(copy number of nuclear DNA/2). Data are presented as mean (SEM) and *P* values correspond to the output of marginal model analyses. The number of samples (*n*) included at each timepoint is indicated under each graph. Wk, week.

Analysis of the change in mtDNA content in a total of 19 substudy subjects revealed a significant increase over 24 weeks [+600 (273.5) copies/cell, *P *= 0.042 at region 1 and +1134.1 (528.4) copies/cell, *P *= 0.046 at region 2, Figure 2] in keeping with the gene expression changes identified.

### Adipose tissue protein

**Figure 3.**
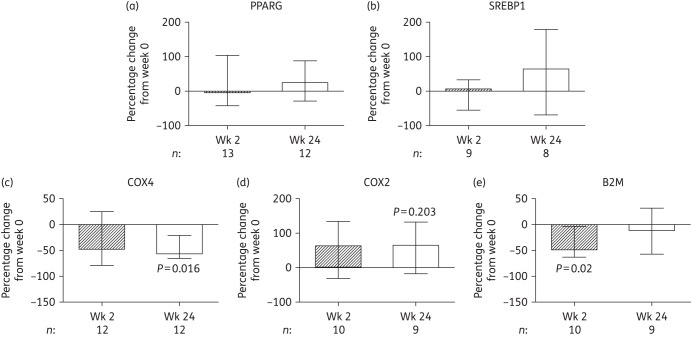
Changes in the expression of proteins in the adipose tissue of subjects initiating HIV PI-only ART. Quantification was by immunoblotting with subsequent densitometric analysis. The bars represent the median (IQR) percentage change from week 0 to week 2 and to week 24. *P* values correspond to the Wilcoxon signed rank test. (a) PPARG, peroxisome proliferator-activated receptor γ. (b) SREBP1, sterol regulatory element binding protein 1. (c) COX4, cytochrome *c* oxidase subunit 4. (d) COX2, mitochondrially encoded cytochrome *c* oxidase subunit 2. (e) B2M, β2 microglobulin. The number of samples (*n*) included at each timepoint is given under each graph. Wk, week.

Overall, biopsies from 16 subjects contributed to protein analyses, although the numbers used in each protein analysis varied depending on sample abundance. No significant change was observed in PPARG and SREBP1 protein (Figure 3a and b), although levels tended to increase. In keeping with gene expression and mtDNA results, improved mitochondrial function was evidenced by decreased levels of nuclear-encoded COX4 at week 24 [−56.3% (−65%, −29.6%), *P *= 0.016, Figure 3c] and by an increasing trend in mitochondrially encoded COX2 at week 24 [+64.1% (+21.2%, +110.9%), *P *= 0.203, Figure 3d], resulting in a significant increase in the COX2/COX4 ratio at week 24 [+288% (+42.2, +621.7), *P *= 0.038], in contrast to the changes observed previously in tNRTI-mediated SAT toxicity.^18,33^ Also observed were significantly reduced levels of the inflammatory marker B2M at week 2 [−48.7% (−60.2%, −22.6%), *P *= 0.02] but not at week 24 (Figure 3e).

### Adipocyte density

**Figure 4.**
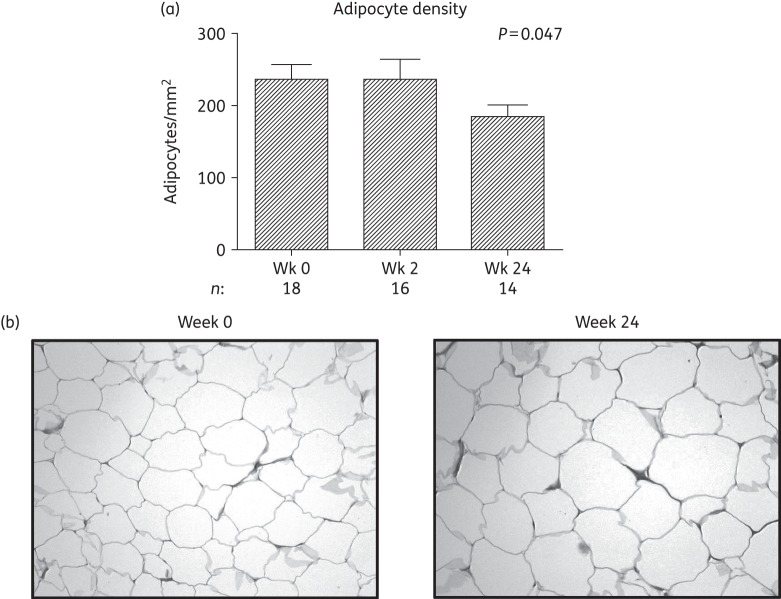
Histological analysis of the adipose tissue biopsies of subjects initiating HIV PI-only ART. (a) Change in adipose tissue adipocyte density over 24 weeks. The number of adipocytes per field (×10 magnification) was quantified using viewing software with adipocyte density expressed as the number of adipocytes/mm^2^. Data are presented as mean (SEM) and *P* values correspond to the output of marginal model analyses; *n *= 18, 18 and 14 at weeks 0, 2 and 24, respectively. Wk, week. (b) Representative micrographs of adipose tissue biopsy sections at week 0 and at week 24 taken at ×10 magnification, stained with haematoxylin phloxine saffron.

Biopsy sections from all 20 subjects contributed to adipocyte density analysis. Histological analysis revealed normal white adipose tissue structure at all timepoints. SAT adipocyte density decreased significantly over 24 weeks [−32.3 (15.5) cells/mm^2^, *P *= 0.047] (Figure 4a), implying larger adipocytes (Figure 4b), which is in keeping with the observed increases in body fat by DXA and CT.

## Discussion

This is the first study (to our knowledge) to explore the *in vivo* effect of PI-only ART initiation on adipose tissue composition and function independently of other ART classes. Our findings suggest improved SAT function as reflected by increases in adipocyte size and molecular changes consistent with improvements in lipid metabolism, mitochondrial function and inflammation with PI-only ART initiation. Such changes indicate a return to health associated with the suppression of HIV replication and do not support the introduction of new toxicity with PI exposure. The use of focused transcriptional profiling, mtDNA, histological and protein analyses in parallel with the assessment of clinical metabolic parameters and body composition allowed a detailed evaluation of the metabolic effects of initiating PI-only ART. Using this approach, adipose tissue toxicity indicative of the development of lipodystrophy was not detected thus challenging the conventional view that PIs cause adipose tissue toxicity.

While the role of tNRTI in the development of SAT toxicity is well established,^9,32^ PIs have also historically been implicated,^7^ with the primary mechanism proposed being the inhibition of *PPARG*, *CEBPA* and *SREBF1* activity resulting in impaired adipogenesis.^10,11^ However, in the current study, initiation of PI-only ART did not lead to either the clinical changes suggestive of lipoatrophy or reductions in expression of these key regulators at either the mRNA or protein level in SAT (Figure 1a and Figure 3a and b). In fact, expression of *PPARG* and its downstream targets, *FABP4* and *LPL*, increased (Figure 1a), suggesting an increase from diminished baseline levels in untreated HIV infection.^4,34^ Moreover, the observed increases in adipocyte size and decreases in the expression of the triglyceride lipase *LIPE* are indicative of increased adipocyte lipid uptake and storage, events that are consistent with increased PPARG activity.

Although our findings contrast with previous clinical studies,^17,18^ subjects in these studies had previous and concomitant exposure to tNRTI, thereby limiting the ability to determine the relative contribution of PI to the observed toxicities. Furthermore, our results are consistent with a recent study comparing two ART combinations (neither of which contained tNRTI) in which SAT adipogenic gene expression increased in subjects initiating PI-containing ART but not with non-PI ART initiation.^35^ These data, coupled with several clinical trials showing an absence of SAT loss with use of PI-containing ART without a tNRTI,^24,36,37^ reinforce our view that the use of PIs is not independently associated with SAT toxicity *in vivo*.

Exposure to certain PIs, including lopinavir/ritonavir, has also been associated with insulin resistance *in vitro*^10,13^ and in healthy volunteers.^30,38^ Despite the transcriptional changes observed suggesting insulin resistance in this study (Figure 1b), there was no effect on systemic insulin sensitivity (HOMA-IR), indicating that the transcriptional changes in SAT were insufficient to affect whole-body insulin sensitivity. Although use of the hyperinsulinaemic–euglycaemic clamp may have allowed more sensitive detection of changes in insulin sensitivity, the findings of previous studies using this method were consistent with ours.^39,40^

Cross-sectional studies have previously demonstrated increased expression of inflammation-related genes concurrent with reduced expression of *PPARG* and PPARG-target genes in the SAT of ART-naive HIV-infected subjects,^4,34^ presumably reflecting the pro-inflammatory effects of uncontrolled HIV infection. In this study, we found that initiation of PI-only ART was associated with the reverse—increased PPARG activity and decreased expression of inflammatory markers *IKBKB* (mRNA) and B2M (protein)—implying a recovery from HIV-induced effects on SAT rather than PI-mediated toxicity. Furthermore, mitochondrial toxicity, another key feature of both untreated HIV and ART-mediated adipose tissue toxicity,^3,9^ was not observed in this study. In fact, initiation of PI-only ART led to improvements in markers of mitochondrial function in SAT; mtDNA and the COX2/COX4 ratio increased while mRNA expression of the stress-responsive regulators of mitochondrial biogenesis *NRF1* and *CRTC3* decreased*.*^41,42^ These outcomes contrast considerably with the mitochondrial dysfunction observed with tNRTI in previous studies,^24,33^ and further support an overall improvement in SAT function with initiation of PI-only ART.

Taken together, our findings suggest that the initiation of PI-only ART, independent of the confounding effects of other ART classes, displays negligible SAT toxicity in clinical, transcriptional, protein and histological analyses. In the context of an ever-expanding number of PLWH and with the need to increase global access to ART,^43^ the safety and efficacy of novel and cheaper ART regimens including PI monotherapy are being increasingly explored.^44^ Within this clinical setting, our results are particularly relevant and reassuring considering the lack of significant PI-mediated SAT toxicity observed.

This study had limitations. The absence of a group initiating alternative ART regimens makes it difficult to differentiate between the effects of PIs on SAT function and the effect of suppressing HIV replication. In addition, the relatively advanced immunosuppression of substudy subjects at baseline may have contributed to an augmented ‘return to health’ phenomenon that could differ from cohorts beginning ART at higher CD4+ counts. However, our molecular and clinical data are consistent with the findings of recent randomized studies that also demonstrate generalized increases in adiposity in subjects of higher baseline CD4+ counts when initiating several types of tNRTI-sparing ART.^45,46^ With follow-up limited to 24 weeks, SAT dysfunction arising from longer periods of PI exposure may not have been detected. Despite this, PIs can induce adipocyte toxicity *in vitro* after as little as 5 days of exposure^10^ and *in vivo* transcriptional changes indicative of SAT toxicity have been demonstrated after even shorter periods of ART exposure.^23,35^ While it may be argued that these results may not apply to the PI class as a whole (only two types of PI were examined), initiation of other commonly used PIs, such as darunavir and atazanavir, has a similar effect on body composition.^47^ Finally, study subjects were exclusively Asian. Nevertheless, there is no reason to suggest that the effects of PI on SAT would be markedly different in other patient groups.

In summary, the initiation of PI-only ART in this study facilitated an improvement rather than a deterioration in SAT function, with the changes observed consistent with a return to health, including increased adiposity, reduced levels of inflammation and improved mitochondrial function in adipose tissue.

## 

### Additional members of the HIVNAT-019 Study Group

Saskia R. Autar, Jasper van der Lugt, Anchalee Avihingsanon, Supalak Klungklang and Sasiwimol Ubolyam.

## Funding

This work was supported by grants from: the European AIDS Treatment Network (NEAT IG3); the Wellcome Trust (097424/Z/11/Z); Molecular Medicine Ireland; Science Foundation Ireland (09/RFP/BMT2461); and Instituto de Salud Carlos III (Spain), cofinanced by FEDER (PI14/00700 and PI14/00063). The original pharmacokinetic study was funded by Roche Pharmaceuticals.

## Transparency declarations

J. C. has received research grants from ViiV Healthcare, Janssen and Merck, and has received honoraria as a speaker for or member of Advisory Boards for ViiV Healthcare, Gilead, MSD, Janssen, Chugai and Novartis. P. D. has received honoraria as a speaker for or member of Advisory Boards for Gilead Sciences, Bristol-Myers Squibb, Abbvie, ViiV Healthcare, Janssen-Cilag and Merck Sharp & Dohme, and has received research grants from Gilead Sciences, Abbvie, Boehringer Ingelheim, Pfizer Inc. and Janssen-Cilag. P. R. through his institution has received independent scientific grant support from Gilead Sciences, Janssen Pharmaceuticals Inc., Merck & Co., Bristol-Myers Squibb and ViiV Healthcare. In addition, P. R. serves on a scientific advisory board for Gilead Sciences, he serves on a data safety monitoring committee for Janssen Pharmaceutica N.V. and he chaired a company-organized scientific symposium for ViiV Healthcare, for which his institution has received remuneration. P. W. G. M. reports grants and personal fees from Janssen-Cilag, grants from GlaxoSmithKline (Ireland), grants and personal fees from Gilead Sciences, grants and personal fees from Bristol-Myers Squibb, grants and personal fees from Merck and personal fees from ViiV, outside of the submitted work. All other authors: none to declare.

### Author contributions

P. P., D. A. C. and J. M. A. L. conceived and designed the main study, contributed to the substudy design and recruited patients to the study. J. C., P. R., P. D., M. G. and P. W. G. M. conceived and designed the substudy. R. T. M. conducted the gene expression experiments. E. R. F. collected the clinical data and performed the mtDNA experiments. E. C. and J. C. carried out the histological analysis. M. G. was responsible for the protein analyses. R. T. M. conducted the statistical analysis and wrote the manuscript. All authors critically reviewed and contributed to the final manuscript.

## Supplementary data

Tables S1 to S3 are available as Supplementary data at *JAC* Online (http://jac.oxfordjournals.org/).

Supplementary Data
