# Supplementary material for: Improved adipose tissue function with initiation of protease inhibitor-only ART
Source: J Antimicrob Chemother. 2016 Aug 11;71(11):3212–21. doi: 10.1093/jac/dkw301 (PMC5079304; doi:10.1093/jac/dkw301)
Supplement: Supplementary Data [file supp_71_11_3212__index.html]

Improved adipose tissue function with initiation of protease inhibitor-only ART — Improved adipose tissue function with initiation of protease inhibitor-only ART — Supplementary Data 

# Improved adipose tissue function with initiation of protease inhibitor-only ART

## Supplementary Data

Supplementary Data

- Supplementary Data - Supplementary Data
